# Machine learning to predict rapid progression of carotid atherosclerosis in patients with impaired glucose tolerance

**DOI:** 10.1186/s13637-016-0049-6

**Published:** 2016-09-05

**Authors:** Xia Hu, Peter D. Reaven, Aramesh Saremi, Ninghao Liu, Mohammad Ali Abbasi, Huan Liu, Raymond Q. Migrino, the ACT NOW Study Investigators

**Affiliations:** 1 Arizona State University, Tempe, AZ USA; 2 Texas A&M University, College Station, TX USA; 3 Phoenix Veterans Affairs Health Care System, Phoenix, AZ USA; 4 University of Arizona College of Medicine-Phoenix, Phoenix, AZ USA

**Keywords:** Machine learning, Atherosclerosis, Diabetes, Prognosis, Model

## Abstract

**Objectives:**

Prediabetes is a major epidemic and is associated with adverse cardio-cerebrovascular outcomes. Early identification of patients who will develop rapid progression of atherosclerosis could be beneficial for improved risk stratification. In this paper, we investigate important factors impacting the prediction, using several machine learning methods, of rapid progression of carotid intima-media thickness in impaired glucose tolerance (IGT) participants.

**Methods:**

In the Actos Now for Prevention of Diabetes (ACT NOW) study, 382 participants with IGT underwent carotid intima-media thickness (CIMT) ultrasound evaluation at baseline and at 15–18 months, and were divided into rapid progressors (RP, *n* = 39, 58 ± 17.5 μM change) and non-rapid progressors (NRP, *n* = 343, 5.8 ± 20 μM change, *p* < 0.001 versus RP). To deal with complex multi-modal data consisting of demographic, clinical, and laboratory variables, we propose a general data-driven framework to investigate the ACT NOW dataset. In particular, we first employed a Fisher Score-based feature selection method to identify the most effective variables and then proposed a probabilistic Bayes-based learning method for the prediction. Comparison of the methods and factors was conducted using area under the receiver operating characteristic curve (AUC) analyses and Brier score.

**Results:**

The experimental results show that the proposed learning methods performed well in identifying or predicting RP. Among the methods, the performance of Naïve Bayes was the best (AUC 0.797, Brier score 0.085) compared to multilayer perceptron (0.729, 0.086) and random forest (0.642, 0.10). The results also show that feature selection has a significant positive impact on the data prediction performance.

**Conclusions:**

By dealing with multi-modal data, the proposed learning methods show effectiveness in predicting prediabetics at risk for rapid atherosclerosis progression. The proposed framework demonstrated utility in outcome prediction in a typical multidimensional clinical dataset with a relatively small number of subjects, extending the potential utility of machine learning approaches beyond extremely large-scale datasets.

## Introduction

Impaired glucose tolerance (IGT) is a risk factor for the development of type 2 diabetes mellitus (T2DM) [1], and both IGT and T2DM are associated with increase in cardio-cerebrovascular related mortality [2, 3]. The Diabetes Epidemiology: Collaborative Analysis of Diagnostic Criteria in Europe (DECODE) [4] study showed a tight correlation between IGT and cardiovascular mortality, and IGT is a known risk factor for early-stage atherosclerosis [5]. In the Actos Now for Prevention of Diabetes (ACT NOW) study, it was shown that pharmacotherapy with pioglitazone in IGT subjects resulted in reduced development of T2DM [6] as well as reduced progression of atherosclerosis [7]. Therefore, identification of IGT subjects who are at risk for rapid atherosclerosis progression, and understanding the important characteristics that affect the identification process, may be beneficial in risk stratification and early intervention. Machine learning (ML) methods have been widely used to learn complex relationships or patterns from data to make accurate predictions [8] and are usually applied in the setting of massive datasets (“big data”). Although encompassing traditional biostatistical approaches such as linear regression modeling, ML approaches, in general, have advantages over traditional frequentist statistical approaches because they can predict patterns without any assumption that simple/complex equations underlie relationships among variables and are able to handle the high-dimensionality nature of medical data [9, 10]. The use of ML approaches in clinical trial data to predict clinical response remains in its infancy. Recently, researchers used data from clinical trials of major depressive disorders (STAR*D and COMED) to predict whether a patient will reach clinical remission from a major depressive episode following treatment with citalopram using stochastic gradient boosting ML approach [11]. Using the data from 768 patients in the Neo-tAnGo chemotherapy clinical trial for breast cancer, ML methods were used to classify cells as cancerous or not [12]. The ACT NOW clinical trial has contributed to novel discoveries on reducing the onset of type 2 diabetes mellitus in at-risk participants using pioglitazone [6] as well as providing insights as to underlying metabolic mechanisms involved with development of diabetes [13–15], but the analytic approaches used involved traditional frequentist biostatistical methods. This study aims to investigate the effectiveness of different ML based methods in predicting IGT patients who will develop rapid carotid atherosclerosis plaque progression in a limited dataset typical of clinical trials.

## Methods

### Study design and subjects

The ACT NOW study design including the exclusion and inclusion criteria have been previously published (Clinicaltrials.gov NCT00220961) [6, 13]. In brief, the ACT NOW study was a multicenter, prospective, randomized, double-blind, placebo-controlled trial to test whether pioglitazone prevents T2DM and progression of carotid intima-media thickness (CIMT) in adults ≥18 years old with IGT (defined by a 2-h plasma glucose concentration of 140–199 mg/dL during a 75 g, 2-h oral glucose tolerance test). Of the 602 total participants, 382 subjects had serial carotid atherosclerosis measurements and comprise the study population of the current study. All research subjects gave informed consent and the study was approved by the Institutional Review Boards at each site.

### Carotid atherosclerosis measurement and progression classification

The method for measurement of carotid atherosclerosis has previously been reported [7]. In brief, all 382 subjects underwent high-resolution B-mode carotid artery ultrasound (Logiq, General Electric, Waukesha, WI) to image the far wall of the right distal common carotid region at baseline and mid-study (15–18 months after baseline). Carotid intima-media thickness (CIMT) was measured, and the absolute difference in CIMT between the two time points was considered the measure of plaque progression (or regression). Subjects with CIMT change in the top decile (*n* = 39, 58.1 ± 17.5 μM change from baseline) were arbitrarily classified as rapid progressors (RP), and the rest (*n* = 343, 5.8 ± 2.0 μM change from baseline, *p* < 0.001 versus RP) were considered non-rapid progressors (NRP). Note that despite the arbitrary nature of the cutoff selection, the CIMT change observed in the RP group (58.1 ± 17.5 μM) represents more than 2 standard deviations of annual CIMT change (11.8 ± 12.8 μM) reported in the Multi-Ethnic Study of Atherosclerosis (MESA) study involving 3441 subjects with multiple cardiovascular disease risk factors [16], providing support for the categorization of this group as rapid progressors.

Demographic, clinical, and laboratory information was collected as previously reported [6, 13] and used as variables for model building.

### Data analytics framework

#### Data analyses settings

##### Notations

Boldface uppercase letters (e.g., **A**) are used to denote matrices, uppercase letters (e.g., A) to denote vectors, and lowercase letters (e.g., a) to denote scalars. The entry at the *i*th row and *j*th column of a matrix **A** is denoted as **A**_ij_. **A**_i∗_ and **A**_∗j_ denote the *i*th row and *j*th column of a matrix **A**, respectively.

Given a set of patients **X** ∈ ℝ^n × d^, *n* is the number of patients and *d* is the number of features. The feature (attribute, variable) vector is denoted as {X_1_, X_2_, …, X_d_}. Let Y ∈ ℝ^n^ be a vector denoting the classes of the patients. In this study, we have two classes for each patient, i.e., Y_i_ used in Hu et al. study [17].

With the notations above, the problem is formally defined as follows: given a set of patients **X** with their class information Y, the aim is to learn a classifier h to automatically assign class labels for unseen patients (i.e., test data).

##### Preprocessing

Data preprocessing was performed to make the input data more consistent to facilitate machine learning algorithms. First, data imputation was performed to deal with missing values. Missing value was crudely imputed as the smallest value for the variable in the dataset. Second, in order to tackle variables with heterogeneous nature, a widely used method [18] was employed to create dummy variables to substitute all possible categories in a categorical variable. Zero or 1 was used to indicate the absence or presence of a categorical variable, thus creating multiple dummy variables for the categorical variable. The number of dummy variables is equal to the number of distinct categories in the original variable.

The variables used in the model are as follows: age; sex; race; Hispanic race; site; family income; randomization to placebo versus pioglitazone; waist circumference; height; systolic/diastolic/mean blood pressure; body mass index; plasma creatinine; urine microalbumin; insulin level; interleukin-6; leptin; plasminogen activator inhibitor-1; C-reactive protein; monocyte chemoattractant protein-1; tumor necrosis factor-1; total cholesterol; triglyceride; low density lipoprotein; alkaline phosphatase; alanine transaminase; aspartate transaminase; hemoglobin; hematocrit; platelet; white blood cell count; and history of hypertension, smoking, the use of alcohol, the use of lipid lowering therapy, the use of nonsteroidal anti-inflammatory medication, the use of angiotensin converting enzyme inhibitor, gestational diabetes, myocardial infarction, stroke, and peripheral vascular disease.

##### Feature selection with Fisher Score

To deal with the multi-modal data consisting of heterogeneous variables, we propose to employ feature selection to first obtain an effective feature space. By introducing feature selection in the learning framework, we exploit its advantages including increased learning performance and computational efficiency, better generalization of the learned model, and interpretability for specific applications. In particular, we employed a supervised feature selection algorithm called Fisher Score in our study. Fisher Score [19], which is one of the most widely used methods, has shown effectiveness in many data mining applications. The basic idea is to select the features that are efficient for discrimination, i.e., feature values of samples within a class are small while being large between classes. The top k features can be obtained with a greedy search method by finding the features with the largest Fisher Scores. Human (clinician) input mainly involved consideration of which redundant/repetitive features are to be discarded (e.g., the presence of hypertension variable and the use of antihypertensive medications variable) and which features are irrelevant to predictive function (e.g., clinical trial variables that were measured after the 18-month outcome has occurred). The investigators were careful in minimizing feature de-selection so as to minimize bias and prevent exlcusion in the model of previously unknown features that could affect the outcome of interest.

##### A probabilistic Bayes model

We employ a probabilistic Bayes model to tackle the classification problem. Bayesian classifiers have been intensively studied to assign the most likely class to a given data instance represented by its feature vector. The classifiers are built upon the Bayes theorem shown as below:

1 $$ P\left(Y\Big|{\mathrm{X}}_1{\mathrm{X}}_2\dots {\mathrm{X}}_d\right)=\frac{P\left({\mathrm{X}}_1{\mathrm{X}}_2\dots {\mathrm{X}}_d\Big|Y\right)P(Y)}{P\left({\mathrm{X}}_1{\mathrm{X}}_2\dots {\mathrm{X}}_d\right)}, $$

where $P\left(Y|{\mathrm{X}}_1{\mathrm{X}}_2\dots {\mathrm{X}}_d\right)$ represents the probability of having class label Y given the data instance $\mathrm{X}=\left\{{\mathrm{X}}_1{\mathrm{X}}_2\dots {\mathrm{X}}_d\right\}$, $P\left({\mathrm{X}}_1{\mathrm{X}}_2\dots {\mathrm{X}}_d\Big|Y\right)$ represents the probability to observe $\mathrm{X}=\left\{{\mathrm{X}}_1{\mathrm{X}}_2\dots {\mathrm{X}}_d\right\}$ in the class Y, $P(Y)$ represents the probability that instances belong to the class Y, $P\left({\mathrm{X}}_1{\mathrm{X}}_2\dots {\mathrm{X}}_d\right)$ is the probability of instance $\mathrm{X}$. To use Bayes theorem for classification, the goal is to find the class, give an instance $\mathrm{X}=\left\{{\mathrm{X}}_1{\mathrm{X}}_2\dots {\mathrm{X}}_d\right\}$, to maximize the posterior probability, shown below:

2 $$ {h}^{*}\left(\boldsymbol{x}\right)= \arg { \max}_cP\left(Y=c\Big|\boldsymbol{x}={\mathrm{X}}_1{\mathrm{X}}_2\dots {\mathrm{X}}_d\right). $$

Since the prior probability $P\left({\mathrm{X}}_1{\mathrm{X}}_2\dots {\mathrm{X}}_d\right)$ is a fixed value in Eq. 1, by substituting Eq. 2 into Eq. 1, it is easy to show that*P*(*Y*|X_1_X_2_ … X_*d*_) ∝ *P*(X_1_X_2_ … X_*d*_|*Y*)*P*(*Y*), indicating that the posterior probability is proportional to likelihood times prior. Therefore, given a data instance $\boldsymbol{x}$, its class label can be determined according to the following Bayes classifier:

3 $$ {h}^{*}\left(\boldsymbol{x}\right)= \arg { \max}_cP\left(\boldsymbol{x} = {\mathrm{X}}_1{\mathrm{X}}_2\dots {\mathrm{X}}_d\Big|Y=c\right)P\left(Y = c\right), $$

which is to maximize the multiplication of likelihood and prior previously discussed. However, the calculation of likelihood $P\left(\boldsymbol{x}={\mathrm{X}}_1{\mathrm{X}}_2\dots {\mathrm{X}}_d\Big|Y=c\right)$ may be difficult especially when the number of data instances is small. To make the computation effective and efficient, a widely used assumption for Bayesian classifiers is that the features are independent with each other given the classes shown as follows:

4 $$ P\left(\boldsymbol{x}={\mathrm{X}}_1{\mathrm{X}}_2\dots {\mathrm{X}}_d\Big|Y=c\right)=P\left({\mathrm{X}}_1\Big|Y=c\right)P\left({\mathrm{X}}_2\Big|Y=c\right)\dots P\left({\mathrm{X}}_d\Big|Y=c\right). $$

The classifier built upon this assumption is Naïve Bayes and while the assumption is simple, Naïve Bayes classifier has shown effectiveness in many real-world applications such as text classification [20] and information retrieval [21]. Naïve Bayes classifier was used in the current study by substituting Eq. 4 into Eq. 3, shown as below:

5 $$ {h}^{*}\left(\boldsymbol{x}\right)= \arg { \max}_c{\displaystyle \prod_i^d}P\left({\mathrm{X}}_i\Big|Y=c\right)P\left(Y=c\right). $$

The proposed method is efficient in terms of training and testing time. Although the real-world dataset the method was used in contains a limited number of subjects, the proposed method has the potential to be applied on a large-scale dataset based on the time complexity analysis as follows: training time of the proposed method is O(|D|Ld + |C||V|), where |D| is the number of instances in the training data, Ld is the average number of variables of a subject in the training data, and |C| is the number of classes and |V| is the number of variables. Testing time of the proposed method is O(|C| Lt), where Lt is the average number of variables of a subject in the testing data.

In addition to Bayesian classifiers, in the pilot study, we also employed another two representative machine learning methods, multilayer perceptron (MLP) and random forest (RF), for classification. MLP is a supervised learning model that uses backpropagation for training an artificial neural network. The learned model consists of multiple layers of nodes, and each layer is fully connected to the next one. The key idea is that, by constructing multiple layers of the model, MLP aims to better map sets of input data onto a set of appropriate outputs. RF is a representative ensemble learning method that constructs a multitude of decision trees for classification. Comparing to traditional decision tree-based learning models, RF is more robust to the overfitting problem and much more effective by combining multiple models. Similarly, RF enjoys the nice properties of decision tree based models such as the interpretability and fast learning rate.

##### Assessment of model performance

The performances of the three ML models were assessed using area under the receiver operating characteristic curve (AUC) and Brier score. The Brier score is a proper score function that measures the accuracy of the probabilistic predictions, with a score of 0 being perfect prediction and a score of 1 being worst score achievable [22]. The AUC was calculated from the probability of RP classification for each subject using each of the learning methods. The Brier score was computed as the mean squared difference between final classification prediction for each subject versus ground truth subject classification [22].

## Results

### Clinical and demographic characteristics

Clinical, demographic, and CIMT data are presented in Table 1. There was no significant difference in age, gender, cardiovascular risk factor co-morbidities, and proportion assigned to pioglitazone between the RP and NRP groups. There were significant differences in enrollment site, proportion with Hispanic race, urine microalbumin, plasma creatinine and serum plasminogen activator inhibitor-1 level between RP and NRP.

**Table 1:** Demographic and clinical and laboratory results

	All	Rapid progressors	Non-rapid progressors	*p* value
	(*n* = 382)	(*n* = 39)	(*n* = 343)	
Age (years)	53.6 ± 0.6	54.1 ± 1.6	53.5 ± 0.6	NS
Female gender (%)	54.19	64.10	53.06	NS
Hispanic race (%)*	31.15	15.38	32.94	0.039
Enrollment site*	382	39	343	<0.001
Site 1	80	5	75	
Site 2	46	15	31	
Site 3	54	1	53	
Site 4	46	7	39	
Site 5	45	8	37	
Site 6	83	3	80	
Site 7	28	0	28	
Hypertension (%)	248 (64.9 %)	21 (53.8 %)	227 (66.2 %)	NS
On lipid lowering therapy (%)	123 (32.4 %)	9 (23.1 %)	114 (33.4 %)	NS
Known vascular disease (%)	7 (1.8 %)	0 (0 %)	7 (2.0 %)	NS
Weight (kg)	92.8 ± 0.9	90.1 ± 2.6	93.1 ± 0.9	NS
Mean arterial pressure (mmHg)	90.7 ± 0.6	90.9 ± 1.6	90.7 ± 0.6	NS
HbA1c (%)	5.48 ± 0.02	5.37 ± 0.05	5.50 ± 0.02	NS
Plasma creatinine*	0.74 ± 0.02	0.87 ± 0.3	0.72 ± 0.03	0.025
Urine mean microalbumin*	14.6 ± 1.0	11.0 ± 2.7	15.0 ± 1.1	0.019
PAI-1*	15.2 ± 0.5	14.7 ± 3.1	15.2 ± 0.4	0.02
Pioglitazone treatment (%)	49.21	46.15	49.56	NS
Baseline CIMT (μM)	759 ± 8	750 ± 29	760 ± 08	NS
Change in CIMT (μM)*	11.1 ± 25.9	58.0 ± 17.5	5.8 ± 17.5	<0.001

*p < 0.05; NS- not significant, HbA1c- glycated hemoglobin, PAI-1-plasminogen activator inhibitor-1, CIMT-carotid intima-media thickness

### Feature selection results

Based on Fisher Scores, the following variables were selected based on the feature selection process: hemoglobin (HGB), mean plasma creatinine (MEAN_PCREAT), PCREAT, gestational diabetes (GDM)_Y_dummy, arterial procedures (OpArtery)_N_dummy, OpArtery_Y_dummy, medical center (CURRENTCENTER), SITE, GDM_N_dummy, Ethnicity_Hispanic (H)_dummy, and HISPANIC, Ethnicity_Non-Hispanic (N)_dummy. However, since CURRENTCENTER and SITE are redundant features, we eliminate SITE from the feature set that are fed into the learning phase. We also removed PCREAT because it is redundant with Mean PCREAT. It also demonstrates the importance of incorporating domain knowledge into the proposed data-driven framework. More sophisticated domain knowledge, such as group structure of the features or pair-wise partial order between some features, could be further incorporated in the framework to improve the learning performance. Since it is beyond the scope of this work, we leave it as future work.

### Learning performance of the baseline methods

We evaluated the performance of several representative learning methods with threefold cross validation. In particular, the data were randomly divided into a training set (67 % of subjects) whose data were used to build the model, and a test set (33 % of subjects) whose data were used to validate the built model. While each of the methods had good performance overall, Naïve Bayes with feature selection achieved the best performance, which resulted in correct classification in 340 of 382 subjects (89.23 %), AUC of 0.797 and Brier score of 0.086 (Table 2).

**Table 2:** Performance of baseline models

Performance parameter	Ml Naïve Bayes with feature selection	Ml Naïve Bayes without feature selection	Multilayer perceptron with feature selection	Multilayer perceptron without feature selection	Random forest with feature selection	Random forest without feature selection
AUC	0.797	0.745	0.711	0.703	0.736	0.703
Correctly classified cases	340 (89.2 %)	290 (75.9 %)	339 (88.7 %)	330 (86.4 %)	338 (88.5 %)	343 (89.8 %)
Incorrectly classified cases	42 (10.8 %)	92 (24.1 %)	43 (11.3 %)	52 (13.6 %)	44 (11.5 %)	39 (10.2 %)
Brier score	0.085	0.222	0.086		0.105	

Also, we investigated the effectiveness of introducing feature selection method in the data analytics framework. The experimental results showed that all of the three methods achieved significantly better results by using feature selection, and Naïve Bayes method achieved AUC of 0.797 (with feature selection) and 0.745 (without feature selection).

## Discussion

The novel finding of our study is that machine learning methods can be applied to a limited dataset typical of a clinical trial in order to predict impaired glucose tolerance subjects who will develop rapid carotid plaque progression with overall good performance. Our results demonstrate the potential utility of sophisticated Bayesian approaches in predicting clinical events from limited clinical datasets.

In 2010, approximately 1 in 3 adults in the USA or about 79 million people had prediabetes [23], which includes IGT and impaired fasting glucose. Aside from the risk for developing diabetes, prediabetes by itself is also independently associated with future risk of stroke [24]. It is therefore critical that we develop tools for early identification of at-risk patients who might benefit from targeted early intervention, both non-pharmacologic and pharmacologic.

The medical field remains almost universally reliant on traditional frequentist low-dimensional statistical approaches for building risk prediction models [9], such as linear and logistic regression models. These approaches are disadvantaged by their reliance on the assumption that simple or complex equations underlie the relationships among variables and the limitations imposed by the high dimensionality of hundreds of features/variables typical of clinical trials or human studies. Machine learning approaches have the potential to overcome these disadvantages. Machine learning is the study of computer algorithms and optimization techniques that can learn complex relationships or patterns from data which in turn can be used to make accurate predictions or decisions [10]. Pattern recognition ML algorithms can be useful for prediction even if no mathematical relationship exists among variables and ML approaches can apply in infinite dimensional spaces. Additionally, the testing of model performance derived from a training set to a separate held-out validation set enhances generalizability of the prediction model allowing for a dynamic ability to learn from new data to optimize the prediction model. Although its current use is predominantly on massive datasets in social media, finance, and information technology business applications, ML may also be useful in high-dimensional but limited dataset (in terms of number of subjects) typical of human studies and clinical trials. In addition, the widespread use of electronic medical records from large health care systems to small independent clinical practices point to an ever-increasing need for novel methods to analyze complex big data. Our results support the use and application of ML approaches to predict outcomes in a limited dataset but with a large number of demographic, clinical, and laboratory variables. It is important to note that even though we used our ML methods in a clinical dataset with a limited number of subjects, we expect the approaches to perform well, if not better, with a large number of subjects. A larger number of subjects (bigger dataset) allows more robust cross validation of model performance to a held-out dataset that would enhance the generalizability of the model. The major problem of clinical trials, however, lies not in too large a sample size but often the opposite, the smaller number of subjects enrolled. This is due to the cost of performing clinical trials plus the ethical mandate of enrolling only the number of subjects that is predicted to statistically show significant differences among treatment options and no more, to ensure research subject safety. Traditional frequentist biostatistical approaches currently being used by the medical community are limited by the dual conditions of small sample size and hyperdimensional datasets typically present in real-world clinical trials, which are conditions that may be ideally addressed by ML approaches, as we have shown in this study.

Among several learning methods used, we found that Naïve Bayes with feature selection performed the best. This is likely because probabilistic Bayesian models perform well with multi-modal data because it assumes independence in inferring probability of each feature. A strong assumption of Naïve Bayes model is that the features are conditionally independent given the label. The assumption may not always hold true for clinical data, but we believe this assumption is reasonable for this study because of the following reasons. First, the conditional independence is a relaxation to enable the calculation of conditional probability, but it is not strictly required for using the model. Naïve Bayes model has been widely used in many real-world problems in which the assumption may not hold, and it achieved better performance in our study compared with other methods. Second, some features in our data, although not all of them, are conditionally independent with each other given the label, e.g., age and gender. A potentially interesting extension of this work and a promising future direction is to investigate how the conditional dependencies can be learned and modeled in the Bayes-based models. The findings motivate us to explore even more sophisticated probabilistic Bayesian models in future work to improve the proposed framework.

We were able to achieve several nice properties by employing feature selection in the data analytics framework. First, we achieved improved performance by introducing feature selection and demonstrated that feature selection has the potential to improve this type of clinical investigation by finding the most effective set of variables. Second, by reducing the number of variables (ten variables in our study), the approach allowed clinicians to manually examine the selected variables and thus improved the interpretability of the learned model. Third, with limited number of selected variables, we could now apply the proposed framework on larger scale datasets that were previously difficult to process with high-dimensional data.

The proposed framework, including preprocessing, feature selection and prediction, is general and can be easily extended to many other data-driven problems in clinical research under some specific conditions. First, a strong assumption in Naïve Bayes based methods is that the features are conditionally independent given the label. To extend the proposed model, we need to have a good understanding of the nature of features. Second, clinician input was incorporated in feature selection for use in the model. Different problems/datasets may require very different domain knowledge to select more informative or useful features. Applying the proposed framework from this initial study to other problems/datasets is potentially important and is one of our future goals.

An important limitation of the study is the inability to determine the generalizability of the ML models derived from the ACT NOW dataset to other prediabetic groups or populations, which should be the focus of future studies looking at real-world performance of ML approaches. This limitation, however, is intrinsic to the nature of clinical trials whose findings or conclusions need to be validated in the general clinical population. Also, given large number of variables before feature selection, it is difficult to model and incorporate domain knowledge from physicians into the framework.

## Conclusions

In conclusion, ML methods were applied to a clinical trial dataset and showed good performance in identifying/predicting impaired glucose tolerance participants who developed rapid carotid plaque progression. Naïve Bayes method showed superior performance over multilayer perceptron and random forest methods and feature selection improved predictive performance. Our findings point to the utility of ML methods in data analytics for clinical applications.
